# Effects of Fiber Shape on Mechanical Properties of Fiber Assemblies

**DOI:** 10.3390/ma16072712

**Published:** 2023-03-29

**Authors:** Dandan Xu, Huibin Ma, Yu Guo

**Affiliations:** 1 College of Mechanical Engineering, Zhejiang University of Technology, Hangzhou 310023, China; xudandan@zjut.edu.cn (D.X.); billmdy@163.com (H.M.); 2 State Key Laboratory of Clean Energy Utilization, Department of Engineering Mechanics, Zhejiang University, Hangzhou 310027, China

**Keywords:** fiber shape, compression, tension, shear, flexible fibers, discrete element method

## Abstract

The effects of fiber shape on the mechanical responses of fiber assemblies under compression, tension, and shear deformations are numerically investigated using the discrete element method (DEM). Simulations of the compression of ring-shaped fibers are consistent with experimental results, verifying the discrete element method code. In the compressive tests of S-shaped fibers, pressure exhibits a nonmonotonic dependence on fiber curvature; while in the tensile tests, yield tensile stress generally decreases with increasing fiber curvature. In the shear tests, yield shear stress decreases with increasing fiber curvature for the S-shaped fibers, and the smallest yield shear stresses and the smallest coordination numbers are obtained for U-shaped and Z-shaped fibers. It is interesting to observe that for the assemblies of various fiber shapes, yield shear stress increases with increasing maximum Feret diameter of the fibers, which characterizes the largest dimension of a fiber between two parallel tangential lines. These novel observations of the effects of fiber shape provide some guidelines for material designs with the fibers.

## 1. Introduction

Fibrous granular materials are ubiquitous in industries of biomass fuels, agricultural products, polymers, textile products, fiber-reinforced composites, filter, and papers. Mechanical responses of fiber assemblies subject to external loads are critical for processing the materials and final outcomes of the products. Thus, previous studies were conducted to understand compression, tension, and shear flows of flexible fibers. Based on the extensive experimental results of fiber compressions, van Wyk [1] and Toll [2] proposed that applied pressure, *P*, and solid volume fraction, *ϕ*, of a fiber bed followed a power law relationship, and the exponent in the law, which is between 3 and 15.5, depended on the random or aligned microstructure of the fiber packings. Such power law relations of *P* and *ϕ* were also confirmed in the numerical simulations [3,4,5]. The simulation results showed that the increase in fiber–fiber friction coefficient caused the rapid rising of the pressure at a smaller solid volume fraction [4]. Coordination number, defined as an average number of contacting neighbors per fiber, and energy per fiber started to rise at a smaller solid volume fraction for the longer fibers than for the short ones [3]. Fiber stiffness had an impact on the compression behaviors [6,7,8,9]. The pressure curves as a function of sample strain became steeper as the fiber stiffness increased [6]. The addition of a small fraction of stiffer fibers to a bed of very flexible fibers could significantly increase the bulk stiffness of fiber bed by orders of magnitude [7]. For random networks of non-identical fibers, the overall network stiffness decreased as the variability of the fiber stiffness increased at constant mean fiber stiffness [8,9]. Smaller compressive loads and larger non-reversible deformation were obtained for plastic fibers rather than for elastic fibers, and it was found that fiber bending plasticity contributed to the smaller loads at early stage of compression and fiber–fiber contact plasticity caused smaller loads at late stage [10]. An increase in fiber–fiber contact stiffness resulted in a larger bulk stiffness of the fiber assembly [11], and the elasto-plastic contact force and adhesion synergistically enhanced the non-reversible deformation of the fiber bed after the compression [12]. In the numerical modeling of agricultural and biomass materials (e.g., crop stems), non-elastic, hysteretic contact forces, and fiber bending deformations should be considered for a more accurate prediction [13,14].

Some special fiber configurations were also considered in the previous studies. As reported by Masse and Poquillon [15], cross-link connections at the contact positions significantly increased the compression stresses and bulk stiffness at the early stage of compression, due to the strong constraints of cross-links on the relative motion between the connected fibers; nevertheless, larger compression stress and bulk stiffness were observed without cross-links at the late stage, which may be relevant to the stronger fiber orientational alignment without cross-links. Bergström et al. [16] and Borodulina et al. [17] observed that the tensile stiffness and tensile strength of a cross-linked fiber network depended on the fiber length distribution and material strength distribution. Persson and Isaksson [18] investigated dynamic fracture of cross-linked fiber networks under tensile loads, and they found that the continuum model was unavailable to describe the dynamic crack growth of the sparse fiber networks due to the very diffuse fracture zone. Li et al. [19] found that the addition of fibers to a spherical particle bed could increase shear strength of the granular material, enhancing the ability to bear larger loads, and a further improvement in the load bearing was achieved by arranging the fibers in a cage-like structure compared to in a random structure. Rodney et al. [20] performed both tension and compression tests on a packing of a self-entangled single long coiled wire, which showed unusual, reversible dilatancy behavior with the Poisson’s ratio below 0 in tension and above 0.5 in compression.

The early work of Discrete Element Method (DEM) studies of flexible fibers included Yamamoto and Matsuoka [21] and Park and Kang [22]. The DEM method was further developed to investigate the compression of fiber assemblies [3,4,5,12,13,14], and microscopic fiber-scale information (fiber deformation, contact force, and local solid volume fraction) could be observed and analyzed. The DEM was also used to investigate shear flows of flexible fibers [23,24]. In dense flows at a constant solid volume fraction [23], steady shear stress increased with increasing friction coefficient, fiber stiffness, and fiber aspect ratio, as larger fiber-fiber contact forces were induced. In dense flows under a constant normal stress [24], the shear stress initially increased with the friction coefficient and then converged to an upper limit when the friction coefficient reached one, while the shear stress was insensitive to the fiber stiffness and fiber–fiber contact stiffness in such normal-stress-controlled flows. 

In the previous studies, the fibers normally possessed the original shape of a straight line. The original shape is referred to as the equilibrium shape of a fiber under no external forces. How the shape of fibers affects mechanical behaviors of fiber assemblies is not yet well understood. However, a good understanding of the effects of fiber shape is crucial for material designs of the fibers, in order to achieve specific properties and functions. In the present work, the DEM method is used to simulate the compression, tension, and shear flows of the elastic fibers of various original shapes, including ring-shape, S-shape, U-shape, and Z-shape. The effects of the original shape of fibers on the bulk mechanical responses and microstructural properties of the fiber assemblies are analyzed based on the simulations results. The present article is organized as follows: The theoretical aspects and governing equations of the DEM-based flexible fiber model are presented in Section 2. The results of compressive tests, tensile tests, and shear tests are discussed in Section 3, Section 4, and Section 5, respectively. At last, the concluding remarks are drawn in Section 6.

## 2. Flexible Fiber Model

**Figure 1:**
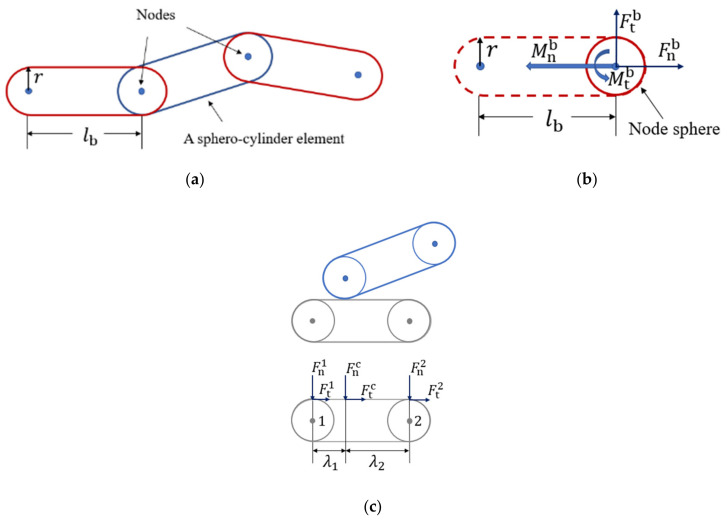
(**a**) A sketch of the flexible fiber model, (**b**) bond forces, and moments exerted on a node sphere, as well as (**c**) contact forces distributed to the two node spheres.

The flexible fiber model developed in previous work [6,10,25] has been used in the present study. As illustrated in Figure 1a, a fiber is discretized into several spherical nodes which are connected by cylinders of the same diameter to the sphere diameter. Virtual bonds are also used to connect the nodes, and they can deform as the fiber deforms. The deformation of bonds leads to the bond forces and moments, which act on the sphere nodes to resist the fiber deformation. The translational and rotational motion of a node sphere is governed by Newton’s second law of motion:

(1) $$m_{i}\frac{dv_{i}}{dt}=F_{ni}^{c}+F_{ti}^{c}+F_{ni}^{b}+F_{ti}^{b}+F_{ni}^{\mathrm{cd}}+F_{ti}^{\mathrm{cd}}+F_{ni}^{\mathrm{bd}}+F_{ti}^{\mathrm{bd}}+m_{i}g,$$

and

(2) $$J_{i}\frac{d\omega_{i}}{dt}=M_{i}^{c}+M_{i}^{b}+M_{i}^{\mathrm{cd}}+M_{i}^{\mathrm{bd}},$$

in which $v_{i}$ and $\omega_{i}$ are the translational and angular velocity vectors (m/s), respectively, of node sphere *i* with mass $m_{i}$ (kg) and moment of inertia $J_{i}$ (kg·m^2^). The translational movement of the node sphere is driven by the normal contact force $F_{ni}^{c}$, tangential contact force $F_{ti}^{c}$, normal bond force $F_{ni}^{b}$, tangential bond force $F_{ti}^{b}$, contact damping forces $F_{ni}^{\mathrm{cd}}$ and $F_{ti}^{\mathrm{cd}}$, bond damping forces $F_{ni}^{\mathrm{bd}}$ and $F_{ti}^{\mathrm{bd}}$, and gravitational force $m_{i}g$. Rotational movement is induced by the moments $M_{i}^{c}$, $M_{i}^{b}$, $M_{i}^{\mathrm{cd}},$ and $M_{i}^{\mathrm{bd}}$ due to the contact forces, bond forces/moments, contact damping forces, and bond damping forces/moments, respectively. In the present fiber model, the basic element is a sphero-cylinder, as shown in Figure 1a. Thus, the contact detection between two fibers is based on the contact between two sphero-cylinders. As demonstrated in the previous work [10], three different contact types exist for a contact between two sphero-cylinders: hemisphere–hemisphere contact, hemisphere–cylinder contact, and cylinder–cylinder contact. The specific, geometry-dependent models of the normal and tangential contact forces, $F_{ni}^{c}$ and $F_{ti}^{c}$, which were proposed in the previous work [10] to calculate the contact forces for each contact type, are utilized in the present simulations. The forces F on the right-hand side of Equation (1) have the unit of N, and the moments M on the right-hand side of Equation (2) have the unit of N·m.

The bond forces and bond moments are functions of bond deformation, which is described by the relative displacements between two connected node spheres. Hence, the normal and tangential bond forces $F_{n}^{b}$ and $F_{t}^{b}$ are expressed as linear functions of normal and tangential displacements $\Delta_{n}^{b}$ and $\Delta_{t}^{b}$, respectively,

(3) $$F_{n}^{b}=\frac{E_{b}A}{l_{b}}\Delta_{n}^{b}=K_{n}^{b}\Delta_{n}^{b},$$

and

(4) $$F_{t}^{b}=\frac{G_{b}A}{l_{b}}\Delta_{t}^{b}=K_{t}^{b}\Delta_{t}^{b}.$$

The bond twisting moment $M_{\mathrm{twist}}^{b}$ and bond bending moment $M_{\mathrm{bend}}^{b}$ are computed incrementally based on the relative twisting angular velocity ${\dot{\theta }}_{\mathrm{twist}}$ and the relative bending angular velocity ${\dot{\theta }}_{\mathrm{bend}}$ between two connected node spheres

(5) $$dM_{\mathrm{twist}}^{b}=\frac{G_{b}I_{p}}{l_{b}}{\dot{\theta }}_{\mathrm{twist}}dt=K_{\mathrm{twist}}^{b}{\dot{\theta }}_{\mathrm{twist}}dt,$$

and

(6) $$dM_{\mathrm{bend}}^{b}=\frac{E_{b}I}{l_{b}}{\dot{\theta }}_{\mathrm{bend}}dt=K_{\mathrm{bend}}^{b}{\dot{\theta }}_{\mathrm{bend}}dt.$$

In Equations (3)–(6), $E_{b}$ and $G_{b}$ ($G_{b}=\frac{E_{b}}{2\left(1+\nu_{b}\right)}$, where $\nu_{b}$ is the Poisson’s ratio of the bond) are the elastic modulus and shear modulus (Pa), respectively, of the bond material; A and $l_{b}$ are the cross-sectional area (m^2^) and length (m), respectively, of the bond; $I=\pi r^{4}/4$ is the area moment of inertia (m^4^); $I_{p}=\pi r^{4}/2$ is the polar area moment of inertia (m^4^); *r* is the radius of the fiber (m); and dt is the time step (s). An illustration of bond forces and moments acting on a node sphere is shown in Figure 1b.

Kinetic energy can be dissipated through deformation and vibration of the flexible fibers. This type of kinetic energy loss is implemented through bond damping forces and moments:

(7) $$F_{n}^{\mathrm{bd}}=\beta_{b}\sqrt{2m_{i}K_{n}^{b}}v_{n}^{r},$$

(8) $$F_{t}^{\mathrm{bd}}=\beta_{b}\sqrt{2m_{i}K_{t}^{b}}v_{t}^{r},$$

(9) $$M_{\mathrm{twist}}^{\mathrm{bd}}=\beta_{b}\sqrt{2J_{i}K_{\mathrm{twist}}^{b}}{\dot{\theta }}_{\mathrm{twist}},$$

and

(10) $$M_{\mathrm{bend}}^{\mathrm{bd}}=\beta_{b}\sqrt{2J_{i}K_{\mathrm{bend}}^{b}}{\dot{\theta }}_{\mathrm{bend}},$$

where $K_{n}^{b}$, $K_{t}^{b}$, $K_{\mathrm{twist}}^{b}$, and $K_{\mathrm{bend}}^{b}$ represent the normal, shear, twisting, and bending stiffnesses (N/m), respectively, of the bond as defined in Equations (3)–(6). The symbols, $v_{n}^{r}$, $v_{t}^{r}$, ${\dot{\theta }}_{\mathrm{twist}}$, and ${\dot{\theta }}_{\mathrm{bend}}$, represent the relative normal velocity, tangential velocity, twisting angular velocity, and bending angular velocity, respectively, between two bonded node spheres of mass, $m_{i}$ and moment of inertia, $J_{i}$. The kinetic energy dissipation rate due to the deformation and vibration of the flexible fibers is determined by the bond damping coefficient, $\beta_{b}$. The larger $\beta_{b}$, the faster the energy is dissipated.

The normal component $F_{n}^{c}$ and tangential component $F_{t}^{c}$ of the contact force $F^{c}$ exerted on a sphero-cylinder element are linearly distributed to the two node spheres of the element, as shown in Figure 1c. The normal and tangential components of the contact force on node sphere 1 can be expressed as

(11) $$F_{n}^{1}=\frac{\lambda_{2}}{\lambda_{1}+\lambda_{2}}F_{n}^{c},$$

and

(12) $$F_{t}^{1}=\frac{\lambda_{2}}{\lambda_{1}+\lambda_{2}}F_{t}^{c},$$

in which $\lambda_{1}$ and $\lambda_{2}$ are the distances between the contact point and the tangent points on the node spheres 1 and 2, respectively. Similarly, the force components on node sphere 2 have the expressions

(13) $$F_{n}^{2}=\frac{\lambda_{1}}{\lambda_{1}+\lambda_{2}}F_{n}^{c},$$

and

(14) $$F_{t}^{2}=\frac{\lambda_{1}}{\lambda_{1}+\lambda_{2}}F_{t}^{c}.$$

The above algorithms were implemented in an in-house DEM code for the modeling of flexible fibers. To verify the code for the originally straight fibers, the uniaxial compression of a packing of cut rubber cords in a cylindrical container was simulated in the previous work [10], and the simulation and experimental results of loading/unloading curves are in good agreement. In the following section, the same DEM code will be validated for the modeling of originally crooked fibers.

## 3. Compression Tests

In this section, the curved fiber model is verified and calibrated against experimental results of compression tests of fiber rings. Thereafter, effect of fiber curvature on bulk compression behaviors is explored based on the simulations of S-shaped fibers.

### 3.1. Fiber Rings

**Figure 2:**
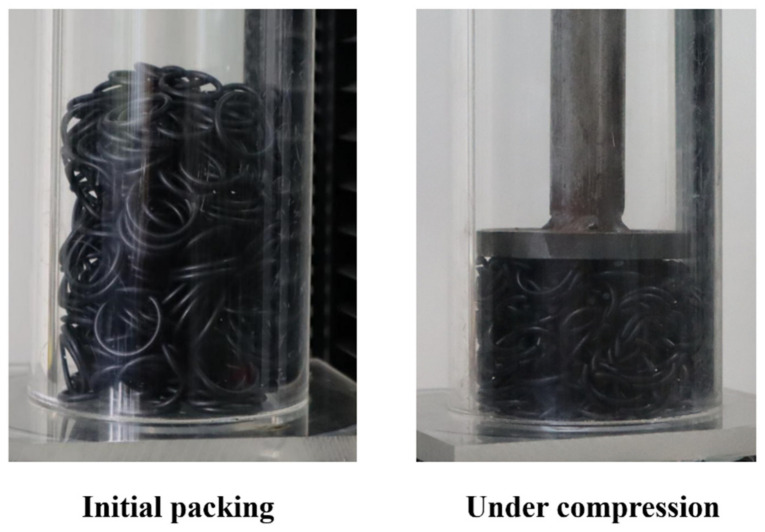
Experimental observations of the packing of flexible rubber rings subject to compression.

To verify the DEM model of the fibers with curvature, experiments and simulations are performed on uniaxial compression of rubber rings. As shown in Figure 2a, a packed bed of 300 rubber rings is formed in a cylindrical container by releasing the rubber rings from the opening of the container one by one. The rubber rings have an outer diameter of circle of 23 mm and a diameter of line of 2.4 mm, a density of 1340 kg/m, and a Poisson’s ratio of 0.5. The cylindrical container made of PMMA has an inner diameter of 80 mm and a height of 200 mm. After the initial packing, a steel punch, the top of which is connected to a ZwickRoell^®^ materials testing machine, moves down at a constant speed of 0.1 m/s into the container to compress the rubber ring bed, as shown in Figure 2b. The load exerted on the punch and the punch displacement are measured by the testing machine during the compression process. 

**Figure 3:**
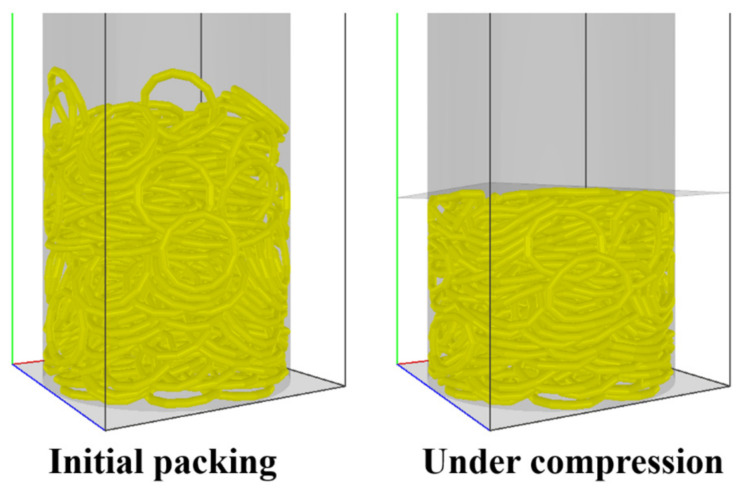
DEM simulation of the uniaxial compression of flexible rubber rings.

**Figure 4:**
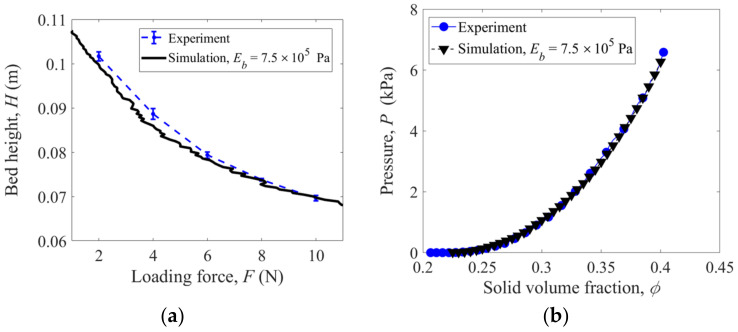
Comparison of experimental and simulation results with rubber rings: (**a**) bed height versus loading force and (**b**) pressure versus solid volume fraction.

The same uniaxial compression of flexible rubber rings is simulated using the present DEM method, as shown in Figure 3, and the parameters used in the simulation are shown in Table 1. By performing a series of simulations with various fiber bending moduli $E_{b}$, it is observed that the DEM simulation results are consistent with the experimental ones when the modulus $E_{b}$ is equal to $7.5\times 10^{5}$ Pa. In the load-controlled compression, as shown in Figure 4a, the height of the granular bed decreases as the loading force increases, while in the displacement-controlled compression, as shown in Figure 4b, the pressure increases exponentially with the solid volume fraction, following a power law relationship like the originally straight fibers [1,2].

### 3.2. S-Shaped Fibers

**Figure 5:**
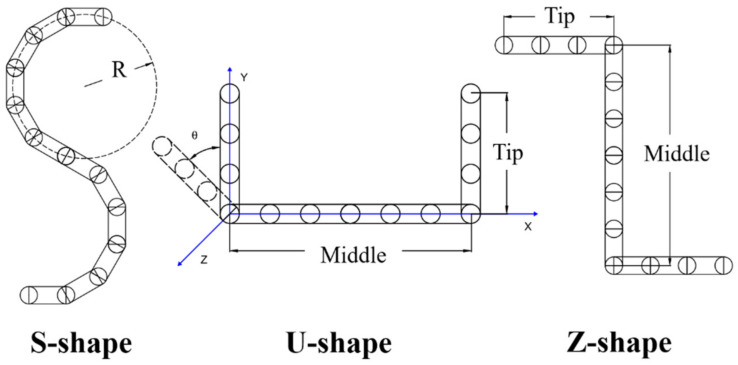
Sketches of S-shaped, U-shaped, and Z-shaped fibers. For the U-shaped and Z-shaped fibers, the length of the middle part is twice the length of each tip.

**Figure 6:**
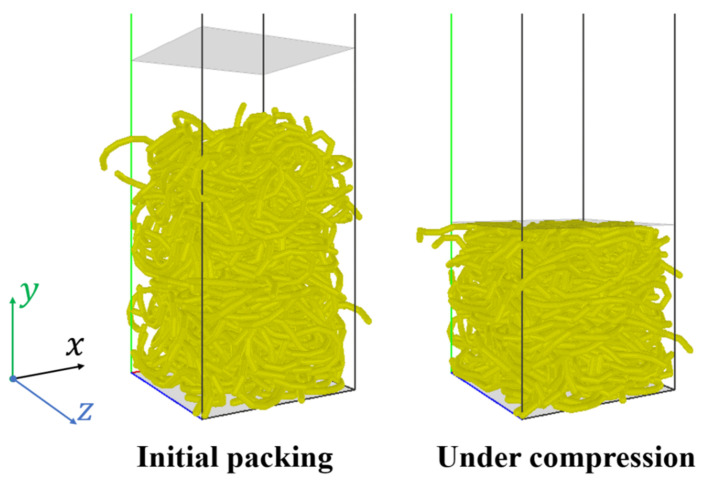
Numerical model of uniaxial compression of S-shaped fibers.

As shown in Figure 5, S-shaped, U-shaped, and Z-shaped fibers are used in the present simulations to explore the effect of fiber shape on the mechanical characteristics of the fiber assemblies. In the simulations of the uniaxial compression of S-shaped fibers, as shown in Figure 6, the periodic boundary conditions are specified in the two horizontal directions (i.e., *x* and *z* directions), and the fibers are deposited under gravity on a flat wall. When the static state is achieved with negligible fiber velocities, the fiber bed is compressed by an upper wall, which moves downwards at a constant velocity of 0.1 m/s. It is noted that the compression results are insensitive to the loading speed in the range 0.01–0.1 m/s according to the present simulation results. The DEM simulation parameters are presented in Table 2. In the simulations, 300 S-shaped fibers of the linear length of 62.83 mm are generated in a rectangular domain of dimensions 60 × 300 × 60 mm^3^. The other properties of the S-shaped fibers are the same to those of the above rubber rings (see Table 1). The curvature of an S-shaped κ is defined as the reciprocal of the radius (i.e., 1/R), as shown in Figure 5. 

**Figure 7:**
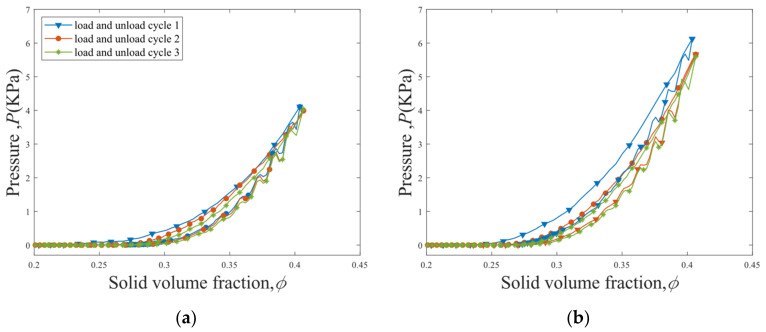
P−ϕ curves for the first three load–unload cycles of (**a**) straight fibers (κ = 0 m^−1^) and (**b**) S-shaped fibers (κ = 100 m^−1^).

The pressure P—solid volume fraction ϕ curves for the first three load–unload cycles of the straight fibers with κ = 0 m^−1^ and S-shaped fibers with κ = 100 m^−1^ are shown in Figure 7. The load–unload cycle 2 shifts to the right-hand side after the cycle 1 due to the fiber rearrangement and the consolidation of the fiber bed after the first load–unload cycle. The P−ϕ loop of the load-unload cycle 3 is very close to that of the cycle 2. At a given solid volume fraction ϕ, a larger pressure P is observed for the S-shaped fibers compared to the straight fibers, and a sharper increase in P with ϕ is obtained for the S-shaped fibers. 

**Figure 8:**
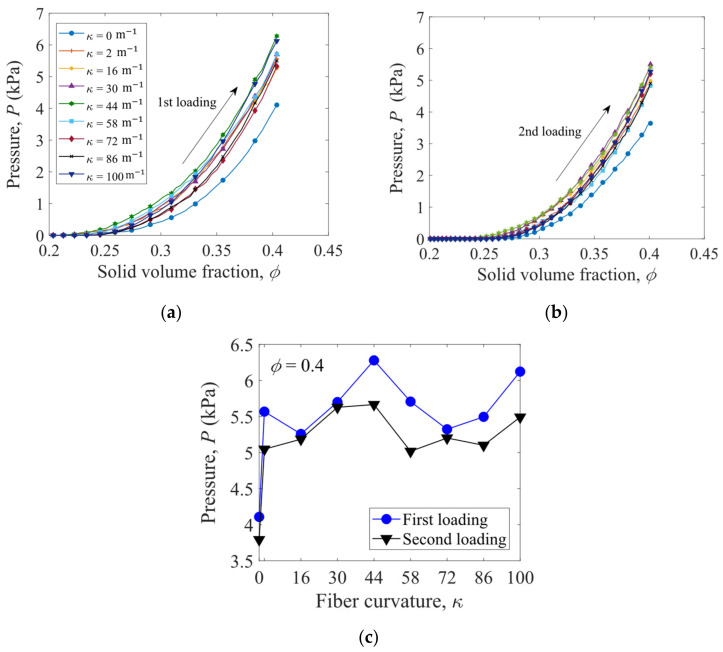
Pressure P versus solid volume fraction ϕ for (**a**) the first loading path and (**b**) the second loading path. The pressure P versus fiber curvature κ at ϕ = 0.4 is plotted in (**c**).

The P−ϕ curves in the first loading path and second loading path for the fibers with various curvatures κ are shown in Figure 8a,b, respectively. The straight fibers have the smallest pressures, and the loading curves of the S-shaped fibers exhibit a non-monotonic dependence on the fiber curvature. As shown in Figure 8c, the peak values of the pressure P at ϕ=0.4 are obtained for the S-shaped fibers with κ = 44 m^−1^ and κ = 100 m^−1^.

**Figure 9:**
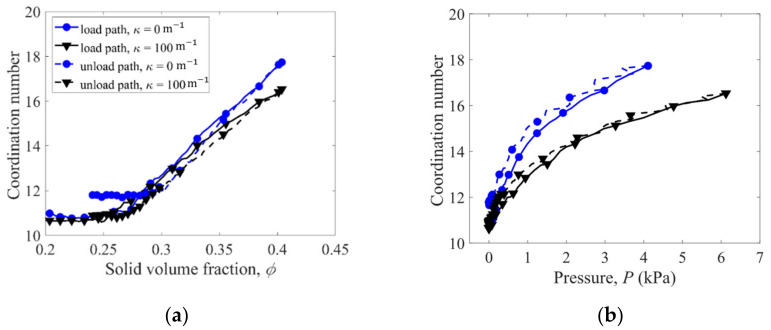
Variation of coordination number with (**a**) solid volume fraction and (**b**) pressure in the load–unload cycle 1.

**Figure 10:**
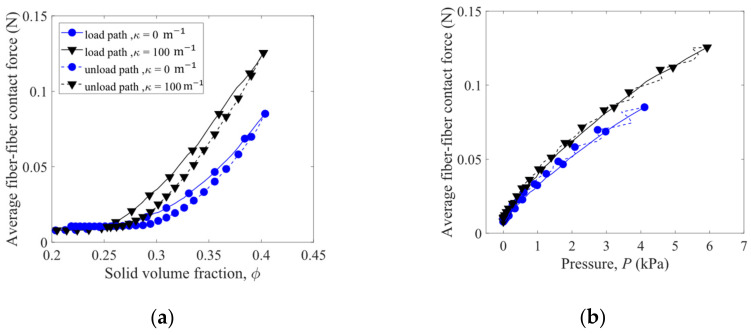
Variation of average fiber–fiber contact force with (**a**) solid volume fraction and (**b**) pressure in the load–unload cycle 1.

In the compression processes, coordination number, defined as the average number of contacting neighbors per fiber, increases with the solid volume fraction ϕ and pressure P, as shown in Figure 9a,b. At a specified ϕ, the coordination number in the load path is greater than that in the unload path (Figure 9a), due to the rearrangement of fibers. while at a specified P, the coordination number in the unload path is slightly greater than that in the load path (Figure 9b). Larger coordination numbers are obtained for the straight fibers with κ = 0 m^−1^ compared to the crooked fibers with κ = 100 m^−1^. However, larger than average fiber–fiber contact forces are obtained for the crooked fibers with κ = 100 m^−1^ (Figure 10), causing larger pressures in the compression of the fibers with κ = 100 m^−1^. Thus, the straight fibers have more but weaker contacts and hence lower pressures in the compression than the crooked fibers. 

**Figure 11:**
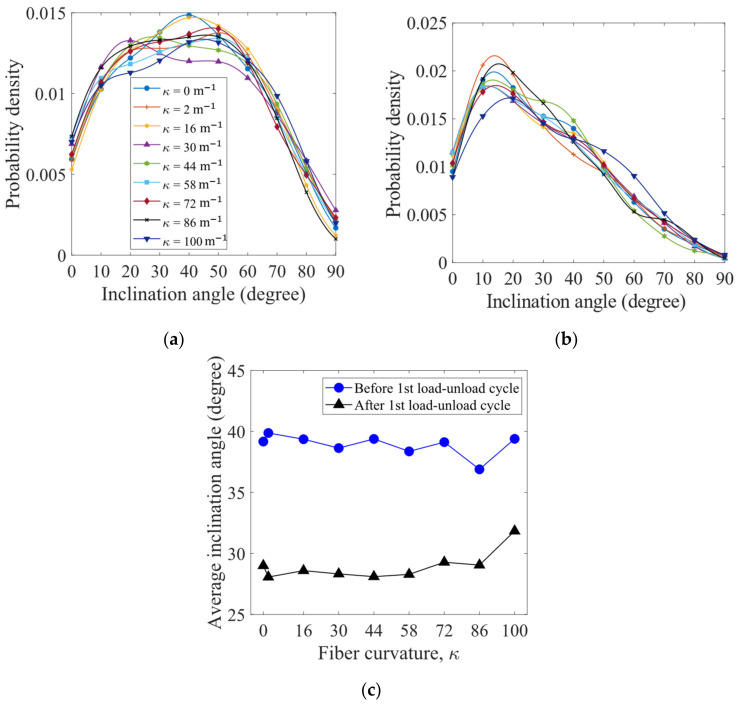
Probability density distributions of fiber inclination angles for the packings (**a**) before and (**b**) after the first load–unload cycle. Average inclination angle as a function of fiber curvature is plotted in (**c**).

To examine the orientation of the fibers, an inclination angle of each fiber is measured as the angle of the line, which connects the two end points of a fiber, from the horizontal plane. The probability density distributions of the fiber inclination angles for the packings before and after the first load–unload cycle are shown in Figure 11a,b, respectively. Before loading, the fibers more likely have the inclination angles between 20° and 60°, while after the first load–unload cycle, the fibers are more likely oriented at the inclination angles between 10° and 20°. The curvatures κ of the fibers have limited impact on the distributions of the inclination angles. As shown in Figure 11c, the average inclination angles are significantly reduced after the first load–unload cycle, indicating that the fibers tend to align horizontally, which gives smaller potential energies of gravity. After the first load–unload cycle of compression, the fibers with the largest curvature considered (κ = 100 m^−1^) exhibit the largest average inclination angle.

## 4. Tensile Tests

**Figure 12:**
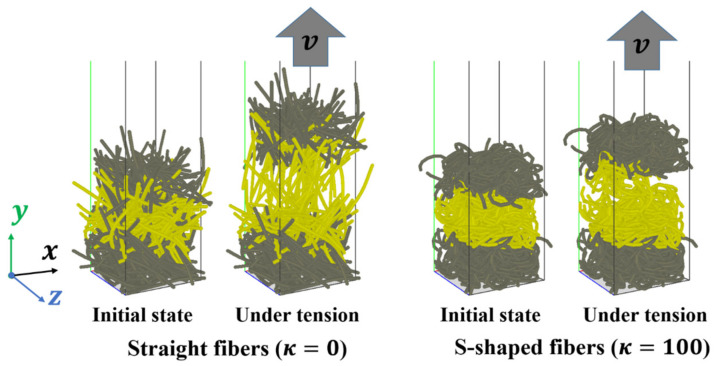
Numerical models of tensile tests.

The assembly of fibers is capable of bearing tensile loads due to strong interlocking and entanglement of the fibrous materials. Therefore, tensions of a bed of 500 fibers subject to stretching loads are also simulated. The DEM simulation parameters are the same to those used in the compression simulations, which are listed in Table 2. As shown in Figure 12, some fibers in the lower region (in gray color) are frozen and remain stationary. Some fibers in the upper region (in gray color), which are also frozen, move upwards in the *y* direction at a constant velocity of 0.1 m/s as a solid body to stretch the fibers in the middle region (in yellow color). In the tensile test simulations, the periodic boundary conditions are assigned in the horizontal *x* and *z* directions. 

**Figure 13:**
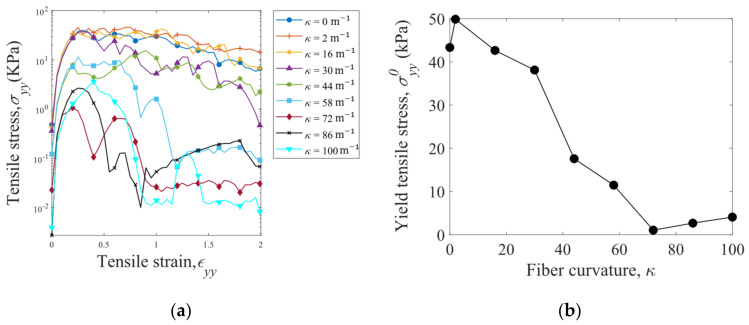
(**a**) Tensile stress $\sigma_{yy}$ versus tensile strain $\epsilon_{yy}$ for the S-shaped fibers with various curvatures κ and (**b**) dependence of yield tensile stress $\sigma_{yy}^{0}$ on the fiber curvature. The normalized fiber assembly sample length is specified as $L_{s}/d$ = 4.17.

Tensile stress $\sigma_{yy}$ is plotted against tensile strain $\varepsilon_{yy}$ in Figure 13a for the fibers with various curvatures κ. In general, the tensile stress initially increases and then decreases until the fiber assembly is torn apart. The peak value of the stress in the $\sigma_{yy}$-$\varepsilon_{yy}$ curve is defined as the yield tensile stress $\sigma_{yy}^{0}$. After the peak $\sigma_{yy}^{0}$, the tensile stress $\sigma_{yy}$ decreases slowly with the strain $\varepsilon_{yy}$ for the fibers with κ≤ 44 m^−1^, and rapid decreases in $\sigma_{yy}$ are observed for the fibers with κ≥ 58 m^−1^. In addition, the yield tensile stress $\sigma_{yy}^{0}$ generally decreases with increasing fiber curvature κ due to reduction in the interlocking of the fibers.

**Figure 14:**
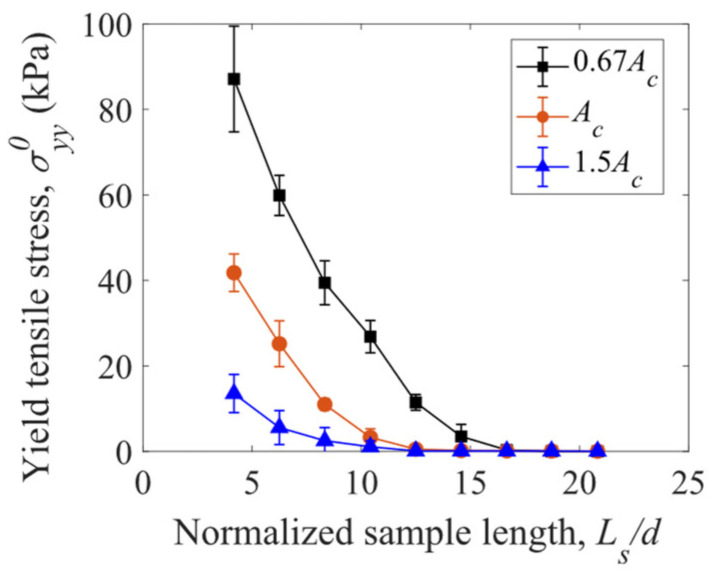
Yield tensile stress versus sample length with various cross-sectional areas of the samples of straight fibers (κ = 0).

The yield tensile stress $\sigma_{yy}^{0}$ is dependent on the length $L_{s}/d$ and cross-sectional area $A_{c}$ of the cubic sample of fiber assembly. As shown in Figure 14, $\sigma_{yy}^{0}$ increases with decreasing sample length $L_{s}/d$ and decreasing cross-sectional area $A_{c}$: the smaller, the stronger. This size effect is due to the fact that more structural defects of weak fiber–fiber connections, which reduce the bulk strength, exist in a larger sample. It is also observed that the assemblies of straight fibers can hardly bear tensile stresses when the normalized sample length is larger than 15.

## 5. Shear Tests

**Figure 15:**
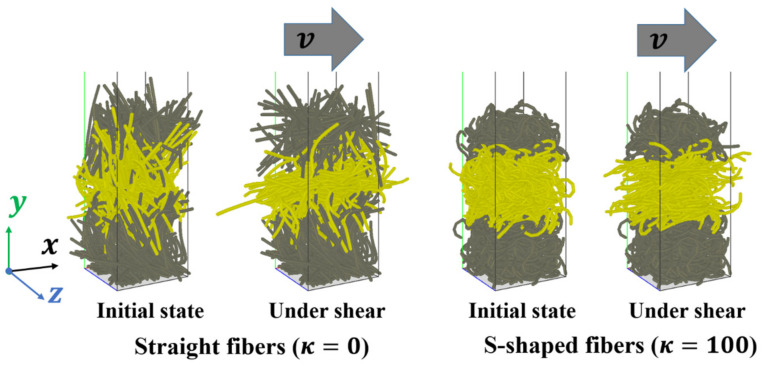
Numerical models of shear tests.

Shear deformations of an assembly of 500 fibers with various fiber shapes are also simulated. The DEM simulation parameters are the same to those used in the compression and tensile simulations, which are listed in Table 2. As shown in Figure 15, some frozen fibers in the lower region (in gray color) remain stationary, and some frozen fibers in the upper region (in gray color) move horizontally in the *x* direction at a constant velocity of 0.1 m/s as a solid body to shear the fibers in the middle region (in yellow color). Periodic boundary conditions are used in the horizontal *x* and *z* directions. 

**Figure 16:**
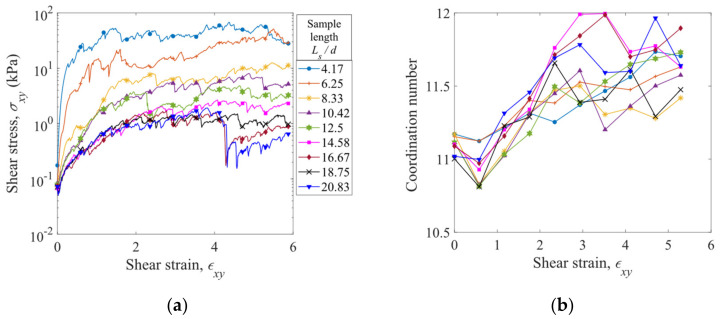
Variations of (**a**) shear stress and (**b**) coordination number with shear strain for the straight fibers with various sample lengths.

The evolution of shear stress $\sigma_{xy}$ with shear strain $\varepsilon_{xy}$ is plotted in Figure 16a for the straight fibers (κ = 0 m^−1^) with various sample lengths. The shear stress increases with the shear strain at the early stage and then fluctuates around a constant level, which is referred to as the steady state. At a given shear strain, the shear stress generally increases as the normalized sample length $L_{s}/d$ is reduced. In the shear process, the coordination number also increases with the shear strain at the early stage and then achieves a constant level, as shown in Figure 16b. Nevertheless, the coordination number, unlike the shear stress, shows a nonmonotonic and irregular dependence on the sample length.

**Figure 17:**
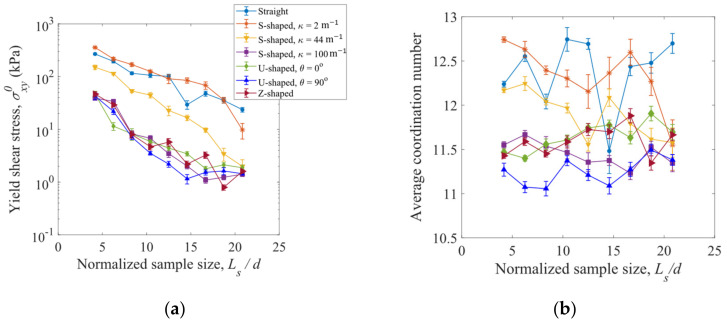
Variation of (**a**) yield shear stress and (**b**) average coordination number with normalized sample size for the fibers of various shapes. The half-length of an error bar represents a standard deviation from the average value at the steady state of shearing deformation.

Yield shear stress $\sigma_{xy}^{0}$, which is defined as the average shear stress at the steady state, is plotted in Figure 17a as a function of the normalized sample length $L_{s}/d$ for the straight fibers, S-shaped fibers with κ = 2, 44, and 100 m^−1^, U-shaped fibers with θ = 0° and 90°, and Z-shaped fibers. Similar to the yield tensile stress (see Figure 14), the yield shear stress $\sigma_{xy}^{0}$ decreases as the sample length $L_{s}/d$ increases. As shown in Figure 17a, for a given sample length, $\sigma_{xy}^{0}$ decreases as the fiber curvature κ increases. The S-shaped fibers with a larger curvature κ = 100 m^−1^, U-shaped fibers, and Z-shaped fibers have the smallest yield shear stresses. The average coordination numbers are insensitive to the normalized sample length, as shown in Figure 17b. In general, the coordination number decreases with increasing fiber curvature κ, and smaller coordination numbers are obtained for the U-shaped and Z-shaped fibers.

**Figure 18:**
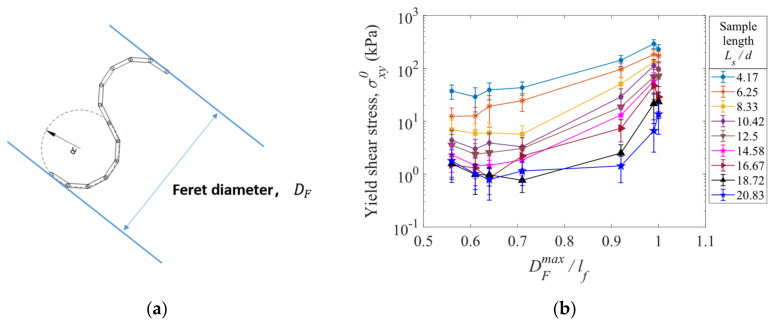
(**a**) An illustration of Feret diameter of a fiber. (**b**) Yield shear stress versus normalized maximum Feret diameter of a fiber, $D_{F}^{\max}/l_{f}$, for various sample lengths. The half-length of an error bar represents a standard deviation from the average value at the steady state of shearing deformation.

A Feret diameter, $D_{F}$, is the distance between a pair of parallel lines which are tangential to the outline of a fiber particle, as shown in Figure 18a. By changing the direction of the parallel lines, different Feret diameters can be obtained for a specified fiber. Thus, a maximum Feret diameter, $D_{F}^{\max}$, exists for a fiber, and it can vary as the fiber shape changes. In Figure 18b, the yield shear stress $\sigma_{xy}^{0}$ is plotted against the normalized maximum Feret diameter, $D_{F}^{\max}/l_{f}$, in which the linear length of a fiber $l_{f}$ is equal to 62.83 mm for all the fibers in the shear deformation simulations. It can be seen that for the fibers of different shapes at various sample lengths, the yields shear stress tends to increase with increasing normalized maximum Feret diameter, $D_{F}^{\max}/l_{f}$. As a result, the shear properties of a fiber assembly are determined by the normalized maximum Feret diameter.

## 6. Conclusions

The numerical models of ring-shaped, S-shaped, and Z-shaped fibers are generated in the discrete element method (DEM) simulations. The mechanical responses of these fiber assemblies subject to compressive, tensile, and shearing loads are numerically investigated. The loading curves of bed height versus loading force (force loading) and pressure versus solid volume fraction (displacement loading) obtained from the compression simulations of rubber rings are consistent with the experimental results, verifying the developed DEM models for the crooked fibers. 

In the compression tests, at a given solid volume fraction, higher pressures are obtained for the S-shaped fibers compared to the straight fibers (>5 kPa versus 4 kPa at solid volume fraction of ϕ = 0.4) due to the larger fiber–fiber contact forces. The effect of fiber curvature on the pressure is non-monotonic. The fiber assemblies can bear tensile forces due to the interlocking and entanglement of the fibers. The yield tensile stress generally decreases (from about 45 kPa to 5 kPa) as the fiber curvature increases (from 0 to 100) due to the reduction in fiber–fiber interlocking with smaller coordination numbers. Larger yield tensile stresses are observed for the samples with shorter length and smaller cross-sectional area, which involve fewer structural defects of weak fiber–fiber connections. 

In the shear tests, the sample size effect also exists in that the smaller the sample length $L_{s}/d$ (<21) is, the larger the shear stresses are. The yield shear stress and coordination number both decrease with increasing fiber curvature κ for the S-shaped fibers, and the smaller yield shear stresses and coordination numbers are obtained for the U-shaped and Z-shaped fibers. The largest dimension of a fiber between two parallel tangential lines, namely the maximum Feret diameter, can be used to characterize the size of a fiber. It is observed that the yield shear stress exhibits an increase with the increase in the maximum Feret diameter for the fibers of various shapes. The yield shear stress can increase by an order as the normalized maximum Feret diameter increases from 0.55 to 1.

The results reported in this work may be useful for estimating how the mechanical responses of the fiber assemblies change when the original fiber shape (the steady shape under no external forces) changes, which can be applied to material designs with the fibers.

## Figures and Tables

**Table 1:** The parameters used in the simulations for the numerical model validation (rubber rings).

Parameters	Values
Fiber shape	Ring
Outer diameter of a fiber ring, $D_{\mathrm{out}}$ (mm)	23
Diameter of fiber line, $d_{s}$ (mm)	2.4
Number of fiber rings, (-)	300
Material density, $\rho_{f}$ (kg/m^3^)	1340
Material Poisson’s ratio, ζ (-)	0.5
Elastic modulus for fiber–fiber contact, $E_{c}$ (Pa)	7.5 ×10^5^
Elastic modulus for fiber bond, $E_{b}$ (Pa)	7.5 ×10^5^
Shear modulus for fiber bond, $G_{b}$ (Pa)	$G_{b}=0.5E_{b}/\left(1+\zeta \right)$
Fiber–fiber friction coefficient, $\mu_{ff}$ (-)	1.4
Contact damping coefficient, $\beta_{c}$ (-)	0.113
Bond damping coefficient, $\beta_{b}$ (-)	3.35 ×10^−2^
Diameter of cylindrical container, (mm)	80
Fiber-cylindrical wall friction coefficient, (-)	0.6
Fiber-flat wall friction coefficient, (-)	0.6
Loading speed, v (m/s)	0.1

**Table 2:** The parameters used in the simulations of compression, tensile, and shear tests.

Parameters	Values
Dimensions of domain, $l_{x}\times l_{y}\times l_{z}$ (mm^3^)	60 ×300 ×60
Fiber shape	S-shape, U-shape, Z-shape
Linear length of a fiber, $l_{f}$ (mm)	62.83
Diameter of fiber line, $d_{s}$ (mm)	2.4
Number of fibers, (-)	300, 500
Material density, $\rho_{f}$ (kg/m^3^)	1340
Material Poisson’s ratio, ζ (-)	0.5
Elastic modulus for fiber–fiber contact, $E_{c}$ (Pa)	7.5 ×10^5^
Elastic modulus for fiber bond, $E_{b}$ (Pa)	7.5 ×10^5^
Shear modulus for fiber bond, $G_{b}$ (Pa)	$G_{b}=0.5E_{b}/\left(1+\zeta \right)$
Fiber-fiber friction coefficient, $\mu_{ff}$ (-)	1.4
Contact damping coefficient, $\beta_{c}$ (-)	0.113
Bond damping coefficient, $\beta_{b}$ (-)	3.35 ×10^−2^
Fiber-flat wall friction coefficient	0.6
Loading speed, v (m/s)	0.1

## Data Availability

The data presented in this study are available on request from the corresponding author.
